# Fluoride And Caring for Children’s Teeth (FACCT): Clinical Fieldwork Protocol

**DOI:** 10.12688/hrbopenres.12799.1

**Published:** 2018-02-28

**Authors:** Patrice James, Mairead Harding, Tara Beecher, Carmel Parnell, Deirdre Browne, Marie Tuohy, Dympna Kavanagh, Denis O'Mullane, Helena Guiney, Michael Cronin, Helen Whelton

**Affiliations:** 1 Oral Health Services Research Centre, University College Cork, Cork, Co. Cork, T12E8YV, Ireland; 2 Cork University Dental School and Hospital, University College Cork, Cork, Co. Cork, T12E8YV, Ireland; 3 Louth Meath Dental Service, Health Service Executive , Navan, Co. Meath, C15RK7Y, Ireland; 4 National Oral Health Office, St. Joseph's Hospital, Mulgrave Street, Limerick, Co. Limerick, V94C8DV, Ireland; 5 South Tipperary, Carlow and Kilkenny Health Service Executive Dental Service, Clonmel Community Care, Clonmel, Co. Tipperary, E91HT96, Ireland; 6 Community Pharmacy, Dental, Optical and Aural Policy Section, Department of Health, Dublin, Co. Dublin, DO2VW90, Ireland; 7 School of Mathematical Sciences, University College Cork, Cork, Co. Cork, T12YN60, Ireland; 8 College of Medicine and Health, University College Cork, Cork, Co. Cork, T12EDK0, Ireland

**Keywords:** water fluoridation, prevention, dental caries, enamel fluorosis, public health, oral health-related quality of life, Dean’s Index, DMF Index, dmf index

## Abstract

**Background: **The reduction in dental caries seen between Irish national surveys of children’s oral health in 1984 and 2002 was accompanied by an increase in the prevalence of enamel fluorosis.  To minimise the risk of enamel fluorosis in Irish children, in 2007, the level of fluoride in drinking water was reduced from 0.8-1.0 ppm to 0.6-0.8 ppm fluoride. Recommendations on the use of fluoride toothpastes in young children were issued in 2002. Fluoride and Caring for Children’s Teeth (FACCT) is a collaborative project between the Oral Health Services Research Centre, University College Cork and the Health Service Executive dental service, with funding from the Health Research Board.

**Aim:** FACCT aims to evaluate the impact and the outcome of the change in community water fluoridation (CWF) policy (2007) on dental caries and enamel fluorosis in Irish schoolchildren, while also considering the change in policy on the use of fluoride toothpastes (2002).

**Methods/Design:** A cross-sectional study with nested longitudinal study will be conducted in school year (SY) 2013-2014 by trained and calibrated dental examiners in primary schools in counties Dublin, Cork and Kerry for a representative sample of children born either prior to or post policy changes; age 12 (born 2001) and age 5, (born 2008). Five-year-olds will be followed-up when they are 8-year-olds (SY 2016-2017). The main explanatory variable will be fluoridation status of the children (lifetime exposure to CWF yes/no). Information about other explanatory variables will be collected via parent (of 5-, 8- and 12-year-olds) and child completed (8- and 12-year-olds only) questionnaires.  The main outcomes will be dental caries (dmf/DMF Index), enamel fluorosis (Dean’s Index) and oral health-related quality of life (OHRQoL). Multivariate regression analyses will be used to determine the impact and outcome of the change in CWF policy on oral health outcomes controlling for other explanatory variables.

## Background

Community water fluoridation (CWF) has been the cornerstone of dental caries (tooth decay) prevention in the Republic of Ireland (RoI) since the 1960s and has played an important role in the improvement in oral health of children ( O'Mullane *et al*., 1986; Parnell *et al*., 2007; Whelton *et al*., 2006) and adults ( Whelton *et al*., 2007). Reviews of the scientific literature have also confirmed the effectiveness of CWF in the control of dental caries ( Australian Government National Health and Medical Research Council, 2007; Centers for Disease Control and Prevention, 2001; Iheozor-Ejiofor *et al*., 2015; McDonagh *et al*., 2000; Rugg-Gunn & Do, 2012). Apart from an increased incidence of enamel fluorosis, reviews of the scientific literature have concluded that there is no credible or definitive evidence linking CWF with negative health outcomes ( Australian Government National Health and Medical Research Council, 2007; McDonagh *et al*., 2000; Sutton *et al*., 2015).

Of the total population of 4.5 million in the RoI, approximately 71% reside in communities served with fluoridated water supplies ( Whelton *et al*., 2006). Fluoride toothpastes, first introduced in the 1970s and in widespread use by the 1980s, now represent 95% of toothpaste sales in most developed countries, including Ireland ( Department of Health and Children, 2002). Hence most people with CWF are exposed to fluoride from water and toothpastes. National surveys of children’s oral health conducted in 1984 ( O'Mullane *et al*., 1986) and 2001–2002 ( Whelton *et al*., 2006) showed an on-going reduction in dental caries levels among 5-, 8-, 12- and 15-year-old lifetime residents in communities with and without CWF. Children with lifetime exposure to CWF had significantly lower dental caries levels than those without. This reduction in dental caries was accompanied by an increase in enamel fluorosis (marks on the teeth associated with fluoride intake during tooth development) levels. In 1984, 6% of 8-year-olds and 5% of 15-year-olds with CWF had some sign of enamel fluorosis. In 2001–2002, using the same measurement criteria, the prevalence of enamel fluorosis was 24% in 8-year-olds and 36% in 15-year-olds. It is important to note that most of the increase occurred in the barely perceptible “Questionable” category of Dean’s Index ( Dean, 1934) and the percentage of children with scores at the higher end of the scale (“Moderate”/“Severe”) was low (1–2%). The prevalence of enamel fluorosis was significantly higher among 8- and 15-year-old children with CWF compared to their counterparts without (p<0.0001) ( Whelton *et al*., 2006).

In response to these findings, in 2002 the Forum on Fluoridation, which was established by the Minister for Health to review CWF in Ireland, issued a number of recommendations aimed at minimising the risk of enamel fluorosis ( Department of Health and Children, 2002). These recommendations included the following:

- In the light of the best available scientific evidence, the Fluoridation of Water Supplies Regulations, 1965 should be amended to redefine the optimal level of fluoride in drinking water from the present level (0.8 to 1.0 ppm) to a level of 0.6 to 0.8 ppm, with a target value of 0.7 ppm. (This recommendation was implemented in 2007).
- Parents should be advised not to use toothpaste when brushing their children’s teeth until the age of 2 years. Prior to this age parents can brush their children's teeth with a toothbrush and tap water. Professional advice on the use of fluoride toothpaste should be sought where a child below 2 years of age is considered to be at high risk of developing dental decay.
- Parents should supervise children aged 2 to 7 years when brushing their teeth and should ensure that only a small pea sized amount of fluoride toothpaste is used and that swallowing of the paste is avoided.

The effect of these major policy changes on children’s oral health will be evaluated as part of the Health Research Board funded Collaborative Applied Research project **F**luoride **A**nd **C**aring for **C**hildren’s **T**eeth (FACCT).

## Aim of the FACCT study

The primary aim of the FACCT study is to evaluate the impact and the outcome of the change in CWF policy (2007) on dental caries and enamel fluorosis in Irish children, while also considering the change in policy on the use of fluoride toothpastes (2002).

This is the protocol for the collection, management and analysis of clinical and questionnaire data for children living in counties Dublin, Cork and Kerry in school year (SY) 2013–2014 (Wave 1) and SY 2016–2017 (Wave 2) to meet the primary aim of the FACCT study.

**Figure 1.**
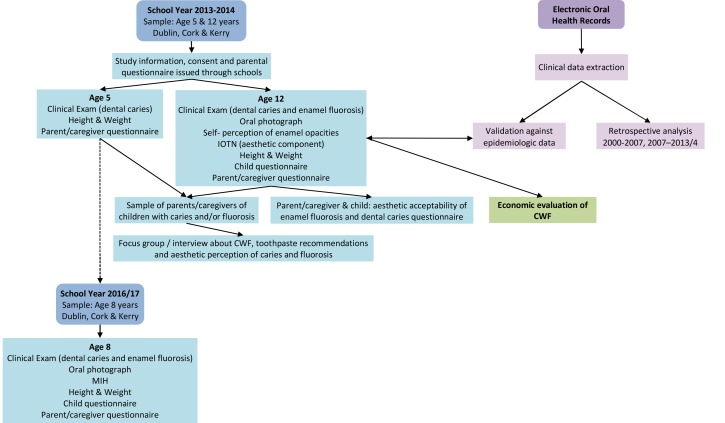
Outline of the FACCT Study. IOTN-index of orthodontic treatment need, CWF-community water fluoridation, MIH-molar-incisor hypomineralisation.

The FACCT study also includes an evaluation of the aesthetic acceptability of enamel fluorosis and dental caries, and the full CARG/2012/34 grant includes an economic evaluation of CWF, and an assessment of the potential of electronic oral health records to generate oral health data that can be used to monitor trends in oral health which will be described elsewhere. An outline of the FACCT study is presented in Figure 1.

## Objectives

1. To measure the level of dental caries in primary teeth of a representative sample of children aged 5 with and without lifetime exposure to CWF in Dublin, Cork and Kerry in SY 2013–2014.
2. To measure the level of dental caries and enamel fluorosis in permanent teeth of a representative sample of children aged 12 with and without lifetime exposure to CWF in Dublin, Cork and Kerry in SY 2013–2014.
3. To re-examine the 5-year-old children when they are aged 8 (SY 2016–2017) to measure the level of dental caries (primary and permanent teeth) and enamel fluorosis (permanent teeth).
4. To assess children’s oral health knowledge, attitudes and behaviour via parental/caregiver questionnaires and self-administered questionnaires (12-year-olds SY 2013–2014; 8-year-olds SY 2016–2017) and to link oral health knowledge, attitudes and behaviour with clinical oral health measures.
5. To compare the level of dental caries in primary teeth of children aged 5 with and without lifetime exposure to CWF (SY 2013–2014) with the level of dental caries recorded in primary teeth of children aged 5 in the corresponding areas in the North South Survey of Children’s Oral Health 2002 ( Whelton *et al*., 2006).
6. To compare the level of dental caries in permanent teeth of children aged 12 with and without lifetime exposure to CWF (SY 2013–2014) with the level of dental caries recorded in permanent teeth of children aged 12 in the corresponding areas in the North South Survey of Children’s Oral Health 2002 ( Whelton *et al*., 2006).
7. To compare the level of dental caries (primary teeth) and enamel fluorosis (permanent teeth) in children aged 8 years with and without lifetime exposure to CWF (SY 2016–2017) with the level of dental caries and enamel fluorosis recorded in children aged 8 in the corresponding areas in the North South Survey of Children’s Oral Health 2002 ( Whelton *et al*., 2006).
8. To examine the relationship between CWF and dental caries in 5- and 12-year old children (SY 2013–2014) and 8-year-old children (SY 2016–2017) in the study areas whilst controlling for other explanatory variables such as age, gender, oral health behaviours and deprivation.
9. To estimate the impact of oral health on the oral health-related quality of life (OHRQoL) of 12-year old children and their parents in SY 2013–2014, and on 8-year-olds and their parents in SY 2016–2017.

### Secondary objective

To measure the weight and height of 5-, 8- and 12-year-olds and compare with BMI measured for the same age groups in 2002 as part of the North South Survey of Children’s Oral Health ( Whelton *et al*., 2006).

## Methods

### Study design

This is a cross-sectional oral health survey of a representative sample of children aged 5 and 12 living in counties Dublin, Cork and Kerry in SY 2013–2014. A nested longitudinal study will be conducted, whereby children who consent to participate in the study at age 5 will be re-examined when they are aged 8 (SY 2016–2017).

The study will compare the primary oral health outcomes dental caries and enamel fluorosis between children with lifetime exposure to CWF (“fluoridated”) and children without lifetime exposure to CWF (“non-fluoridated”).

The specific age groups are chosen to correspond with the WHO index ages for population surveys ( World Health Organisation, 2013) and to allow back comparisons with data from previous Irish oral health surveys.

Children aged 5 in SY 2013–2014 (and aged 8 in SY 2016–2017) were born in 2008–2009 subsequent to both the introduction of recommendations on the use of fluoride toothpastes in young children (2002) and the reduction in the level of fluoride in piped public water supplies (2007), and thus have had full exposure to both policy changes since birth.

Children aged 12 in SY 2013–2014 were born in 2001–2002 at the time that recommendations on the use of fluoride toothpastes in young children were being introduced (2002) and 6 years before the reduction in the level of fluoride in public piped water supplies (2007). Thus children living in areas with CWF throughout their lives had partial exposure to both policy changes. Their permanent teeth mineralised with water fluoride at the higherlevel of 0.8–1ppm F but their permanent teeth were exposed to a reduced topical effect from approximately age 6 onwards.

***Study location***. The study will be conducted in counties Dublin, Cork and Kerry in the Republic of Ireland. Dublin was selected because over a quarter (28%) of the population of Ireland resides in Dublin ( Central Statistics Office, 2011) and it is served by large water treatment plants with an excellent record of control of CWF at the recommended levels. CWF is provided throughout Dublin. Cork and Kerry were selected to represent areas with and without CWF.

### Ethical approval

Ethical approval for the training and calibration of dental examining teams and for data collection, will be obtained from the Clinical Research Ethics Committee of the Cork Teaching Hospitals, prior to the commencement of the study. Informed consent will be obtained from the parents/guardians of participating children and assent from 8- and 12-year-old children.

### Participants

Children in Junior Infants (aged 4–6 years) and 6 ^th^ class (aged 11–13 years) in SY 2013–2014 attending randomly selected primary schools in counties Dublin, Cork and Kerry will be eligible to participate in the study.

***Selection of schools***. The sample of schools will be drawn using stratified cluster random sampling. A list of primary schools in Dublin, Cork and Kerry in SY 2011–2012 (the most recent data available at the time the sample is drawn) will form the sampling frame for the study. Schools for children with special educational needs will not be included in the sampling frame. All schools in Dublin receive a fluoridated water supply. For the Cork and Kerry schools, on the basis that children attending a school with a non-fluoridated water supply are more likely to live in a household without CWF, the sampling frame will be stratified by the fluoridation status of the school water supply, classified as fluoridated or non-fluoridated by the Principal Dental Officers of the Cork and Kerry regions. The sampling frame for Dublin, Cork and Kerry will be stratified by community care area/dental area (n=11: 6 in Dublin, 5 in Cork/Kerry) and school size (small, medium and large). The use of community care/dental area as the smallest geographic unit for sampling replicates the sampling method used in the North South Survey of Children’s Oral Health in 2002 ( Whelton *et al*., 2006) and will facilitate direct comparison between the two data sets. Randomly selected schools with an even distribution of gender will be approached to participate in the study.

Each selected school will be contacted, informed about the study and their willingness to participate ascertained. Where schools decline participation, a replacement school of the same size and fluoridation status will be selected within the same community care area/dental area.

***Sample size***. The sample size calculation is based on prevalence and central tendency data for Dublin, Cork and Kerry obtained in the North South Survey of Children’s Oral Health 2002 ( Whelton *et al*., 2006).

*5-year-olds*

Sample sizes of 5-year-olds will have in excess of 80% power, at a 5% level of significance, to demonstrate that:

- Mean dental caries levels are at least 20% lower in children with lifetime exposure to CWF compared with children without lifetime exposure to CWF living in Cork/Kerry in 2013–2014.
- In Cork/Kerry, the proportions of 8-year-old children (2016–2017) with lifetime exposure to CWF with no enamel fluorosis has increased by at least 5% from 2002.
- In Dublin, the proportion of 8-year-old children (2016–2017) with no enamel fluorosis has increased by at least 5.25% from 2002. Furthermore, the sample size will allow an estimate of the proportion of 8-year-old children with no enamel fluorosis to within 2.5%.

*12-year-olds*

Sample sizes of 12-year-olds in Cork/Kerry will have in excess of 80% power, at a 5% level of significance, to demonstrate that:

- Mean dental caries levels are at least 20% lower in children with lifetime exposure to CWF compared with children without lifetime exposure to CWF living in Cork/Kerry in 2013–2014.
- Sample sizes of 12-year-olds for Dublin will have sufficient precision to estimate caries levels in 2013–2014.

*Adjustments to the sample size*

The sample size is inflated by 40% to account for the design effect due to using a cluster sampling technique. As the 5-year-olds will be re-examined at age 8, the 5-year-old sample is further adjusted to account for loss to follow-up during the subsequent three years (20%). The sample is also adjusted to account for participants being classified as neither “fluoridated” nor “non-fluoridated” (20%) and for non-response (35%) ( Table 1).

**Table 1.** FACCT sample size adjustments.

Age	Region	Fluoridation Status	n	Design effect + 40%	Loss to follow up 20%	Neither F nor NF 20%	Non-response 35%	Final n to issue consents to
**5**	**Dublin**	**Full**	497	696	870	1087	1673	**1673**
	**Cork/Kerry**	**Full**	424	594	742	928	1427	**1427**
		**Non**	424	594	742	928	1427	**1427**
**12**	**Dublin**	**Full**	432	605	605	756	1163	**1163**
	**Cork/Kerry**	**Full**	442	619	619	774	1190	**1190**
		**Non**	442	619	619	774	1190	**1190**
**TOTAL**			**2661**	**3725**	**4196**	**5245**	**8070**	**8070**

***Selection of children within schools***

*Schools with a fluoridated water supply*

The number of boys and girls in schools with a fluoridated water supply to be issued consent forms will be decided *a priori* by the study statistician (MC). These children will be randomly selected within these schools by the examining teams using a random number generating software programme. In instances where there are a number of different classes within one school year (e.g. four different 6 ^th^ classes in the same school), a class will be randomly selected. If insufficient numbers of children are present in the first class selected, another class will be randomly selected until the required number of children to issue consent forms is obtained. Children who are not selected to participate will receive a letter to advise parents/caregivers about the random selection process and to inform them that their child was not selected.

*Schools with a non-fluoridated water supply*

The total number of 5- and 12-year-old children attending schools with a non-fluoridated water supply in SY 2011–2012 is very close to the actual number required to meet the non-fluoridated sample size, therefore examining teams will issue consent packs to all eligible children attending these schools.

### Consent and study documents

Each child who is selected to participate will be given a FACCT study pack to take home to their parents/caregivers. The pack will contain the following:

- **Study Information leaflet**
- **Parent/guardian consent and child assent form**
- **A parent questionnaire** comprising:
  - a residential history and type of water supply at each address (to establish if the child has had lifelong exposure to fluoridated or non-fluoridated water supplies)
  - a brief child medical history to ensure suitability for examination
  - a questionnaire about the child’s oral health behaviours and demographic details
  - Short form of the Parental-Caregivers Perceptions Questionnaire (P-CPQ) ( Jokovic *et al*., 2003; Thomson *et al*., 2013)
- In Cork/Kerry a small **water bottle** with instructions about returning a home tap water sample will be included in the pack

Copies of FACCT information leaflets, consent/assent forms, residential history forms and parent/caregiver questionnaires for 2013–2014 and 2016–2017 are available in Supplementary File 1.

The consent forms and questionnaires will be piloted with parents of children in local Cork schools (representing a spectrum of socio-economic groups), to assess content, terminology, presentation, clarity and respondent burden, and will be amended accordingly. The parent/caregiver questionnaires will be colour coded by age group to easily distinguish between the different age groups in the study. Each document in the consent pack will be labelled with a unique subject number barcode; this barcode will serve to match each subject’s consent form and parent/caregiver questionnaire. The subject number barcode will be assigned centrally by the Oral Health Services Research Centre (OHSRC). Contact details for the OHSRC and the web address for the FACCT study will be provided on the study information leaflet so that parents can clarify any aspects of the study before deciding whether to consent.

Parents/guardians who permit their child to participate in the study will return the completed consent/assent form, medical history form, residential history form and questionnaire to the school in the envelope provided. Returned documents will be evaluated by the examining team for positive consent/assent and to ensure that there is no medical reason to exclude any child. 

Eligible children whose parents/guardians return a completed consent/assent form and a completed medical history form will be included in the study. Eligible children whose parents do not consent or who do not assent in writing (8- and 12-year-olds only), or who are unable or unwilling to participate on the day (all age groups) will not be examined. Children with any medical condition that would mean their participation in the study would pose a risk to their health or wellbeing will also be excluded.

Returning incomplete paperwork will not preclude inclusion in the study provided that the consent/assent form and medical history form are completed. Every effort will be made to obtain missing residential history information via follow-up phone calls with parents/caregivers (who consent to be contacted by the study team). Children whose parents/caregivers do not complete or partially complete the parent survey will be included in the study; however, the child’s oral health data may consequently be excluded from some of the statistical analyses. 

Paperwork and questionnaires returned by parents/caregivers of 5-year-old children who are not clinically examined (because they are absent on the day the examining team visit the school or because the child refuses to be examined on the day) will be retained and we will attempt to follow-up these children when they are aged 8 in SY 2016–2017.

***Translation of study documents***. The Irish language is the first official language of Ireland and the Official Languages Act 2003 promotes its use for official purposes. Therefore, all study documents will be translated into Irish by Ionad na Gaeilge Labhartha (University College Cork) and made available to parents/caregivers and participating children during the study.

A number of validated instruments will be translated into Irish for use in the study. In the case of these validated instruments (Parental-Caregivers Perceptions Questionnaire-P-CPQ (short form; ( Jokovic *et al*., 2003; Thomson *et al*., 2013)), Child Perceptions Questionnaire for children aged 8 to 10 years old – CPQ _8–10_ (long form; ( Jokovic *et al*., 2004)), and Child Perceptions Questionnaire for children aged 11 to 14 years old – CPQ _11–14 _ (short form; ( Foster Page *et al*., 2008; Jokovic *et al*., 2006)) a safeguard will be introduced to ensure the meaning of the validated instruments will be unchanged by the translation into Irish. The translated instruments will be back-translated into English by a second professional translator blind to the original English version. The original English versions and the back translated English versions will then be compared. The Irish wording will be adjusted where necessary to ensure that the meaning of the validated instruments is unchanged by the translation process.

***Data protection***. UCC is the data controller for all data collected in the course of this study. The study will comply with the requirements of the Data Protection Act 1988 and the Data Protection (Amendment) Act 2003. A detailed submission will be made to the Office of the Data Protection Commissioner (ODPC) in relation to management of sensitive personal data collected as part of the FACCT study.

### Training of examining teams

The examining teams will be trained in advance of the fieldwork. Dentists and dental nurses will be instructed in the overall design of the study and in the procedures involved in organising and conducting oral epidemiological fieldwork. A video resource explaining the procedures involved in organising and conducting oral epidemiological fieldwork will be produced and made available to assist the examining teams. Dental nurses will be trained to use direct data entry software on dedicated FACCT clinical laptops. Dentists will be trained in a series of small group interactive teaching sessions based around epidemiological measurement of dental caries, enamel fluorosis and developmental defects of enamel, including discussion of photographic images of enamel fluorosis. The dentists’ training in recognising and scoring enamel fluorosis with Dean’s Index ( Dean, 1934) will be supplemented by an online fluorosis training tool ( Whelton *et al*., 2014). Further school-based training and calibration to benchmark examiners will be undertaken in a local Cork school, involving clinical examination of children.

Manuals will be produced in 2013–2014 and 2016–2017 to assist the examining teams to organise and conduct the oral epidemiological fieldwork (The manuals are available in Supplementary File 2).

Inter-examiner agreement will be determined using Kappa statistics and percentage agreement (Po). The arbitrary divisions provided by Landis & Koch (1977) will be used as a guide in interpreting the Kappa statistics. Dentists will be required to achieve Kappa values above 0.6 indicating at least substantial agreement with the benchmark examiner for the dmf/DMF Index, Dean’s Index and the DDE (developmental defects of enamel) Index before commencing clinical fieldwork.

### Data collection

***Parent/caregiver questionnaires***

*Oral health behaviours and socio-demographic factors*

The parent/caregiver questionnaires will be used to collect information about other explanatory variables, such as children’s oral health behaviours and socio-demographic factors and are available in Supplementary File 1. The questionnaires will include questions about age of commencing toothbrushing with fluoride toothpaste, frequency of brushing and amount of toothpaste used at different points in the child’s life (under 2 years, at 2–3 years and at the time of the survey). Parents/caregivers will also be asked about use of other fluoride modalities such as fluoride mouthrinse and fluoride tablets or drops. Information will be collected regarding snacking frequency and frequency of intake of sugar sweetened beverages. Parents/caregivers will be asked about infant feeding practices, type of infant formula used and source of water used to make up infant formula. Additional questions will be included regarding parental perceptions about the appearance of their child’s teeth and dental attendance. Responses to questions about healthcare cover and parental education will be used to determine a measure of socio-economic status to be used in the analysis.

*Parental-Caregivers Perceptions Questionnaire (P-CPQ)*

Included in the FACCT parent/caregiver questionnaire is the short form version of the Parental-Caregiver Perceptions Questionnaire for children aged 6 to 14 years old (P-CPQ _6–14_) ( Jokovic *et al*., 2003; Thomson *et al*., 2013) ( Supplementary File 1).

***Child oral health-related quality of life (COHRQoL) and oral health behaviours questionnaire***. Twelve-year-olds (SY 2013–2014) will complete a child oral health-related quality of life questionnaire (CPQ _11–14_ (short form) ( Foster Page *et al*., 2008; Jokovic *et al*., 2006) and oral health behaviours questionnaire. Eight-year-olds (SY 2016–2017) will complete a child oral health-related quality of life questionnaire (CPQ _8–10_ (long form) ( Jokovic *et al*., 2004) and oral health behaviours questionnaire. The questionnaires will be completed on a dedicated secure laptop computer prior to the children’s dental examination. Electronic versions of each questionnaire will be created using Microsoft Access, in English and Irish. For 8-year-olds, the questionnaire will be accompanied by a voice-over to reduce the likelihood of difficulties associated with low literacy.

***Clinical examination***. Eligible children will be examined in their school by a trained and calibrated dentist assisted by a trained dental nurse. A fieldwork manual ( Supplementary File 2) will be available to assist examining teams in conducting the clinical examinations. School visits will be arranged at a time that suits the school staff. Clinical data will be entered directly onto a dedicated laptop computer using a software package designed for the North South Survey of Children’s Oral Health ( Whelton *et al*., 2006) and modified for this study. The examining team will confirm the child’s name, signed parent/guardian consent and child’s willingness to take part in the study. A new clinical record will be created in the direct data entry system, and the child’s unique subject number barcode – which has been assigned centrally by the OHSRC and appears on the child’s consent/assent form – will be scanned by a member of the examining team.

The dental nurse will enter the child’s first and surname, gender, date of birth (from the consent form) and will also ask the child’s age. The school name, school roll number, examiner ID and dental nurse ID numbers will also be entered before the clinical examination commences.

*Sequence of clinical examination*

The clinical examinations for each age group will be conducted in a particular sequence as described in Table 2.

**Table 2.** Planned clinical examinations for each age group. DDE - developmental defects of enamel, IOTN- index of orthodontic treatment need, MIH-molar-incisor hypomineralisation.

Sequence	Age Group (School Year)		
	5-Year-Olds (2013–2014)	8-Year-Olds (2016–2017)	12-Year-Olds (2013–2014)
1	Height and Weight	Height and Weight	Height and Weight
2	Dental caries	DDE	Self-perception of enamel opacities
3		Dean’s Index	DDE
4		MIH	Dean’s Index
5		Oral photographs	Oral photographs
6		Dental caries	IOTN– aesthetic component
7			Dental caries

*Dental caries*

Dental caries will be measured in 5-year-old children initially in 2013–2014 (primary teeth) and in the primary and permanent teeth of the same children when they are aged 8 in 2016–2017. Dental caries will be measured in the permanent teeth of 12-year-old children in 2013–2014. The dental caries examination will be performed in a similar manner to the procedure used in the North South Survey of Children’s Oral Health 2002 ( Whelton *et al*., 2006) by a trained and calibrated dentist. Data will be entered by a trained dental nurse onto a dedicated clinical laptop computer.

Each child will lie on a table covered with a foam camping mat, with a clean disposable head rest cover under their head. The teeth will be examined wet, and a WHO Community Periodontal Index (CPI) probe ( World Health Organisation, 2013) will be used to remove plaque and food debris and/or to confirm cavitation. Lighting will be provided by portable dental lights (Daray lights) which were used in previous Irish regional and national oral health surveys. Dental caries will be measured at the dentine level of involvement (d _3c_mft/s and D _3c_MFT/S), according to WHO examination criteria ( World Health Organisation, 2013). These criteria will be expanded to include coding for non-cavitated dentinal caries, where caries is visible as a shadow under the enamel but does not have a detectable cavity through the enamel (d _3vc_mft/s and D _3vc_MFT/S, ‘v’ indicating ‘visual’ caries). Tooth surfaces with fixed braces or bands (i.e. banded/covered tooth surfaces) will be excluded from this examination. Parents of children for whom an urgent need for dental treatment is noted will be notified by the examining team.

*Enamel fluorosis*

**Dean’s index**

Enamel fluorosis will be recorded in the permanent teeth of 8-year-olds (2016–2017) and 12-year-olds (2013–2014) using Dean’s index ( Dean, 1934).

Dean’s index records fluorosis at the level of the subject, i.e. it is a whole mouth score. The procedure used to record Dean’s Index is the same as that used in the 1984 and 2001–2002 national surveys of children’s oral health ( O'Mullane *et al*., 1986; Whelton *et al*., 2006). The child will be examined in the upright position, facing natural light, with the examiner facing the child with his or her back to the light (window). Teeth with orthodontic bands or brackets will be excluded.

All teeth will be examined for fluorosis, and if fluorosis is present, Dean’s Index will be scored on the condition of the two most severely affected teeth. If the two teeth are not equally affected, the less affected tooth will be scored.

**Oral photographs of incisor teeth**

Assessment of fluorosis using photographs addresses the subjective nature of clinical assessment of fluorosis, by allowing the assessment to be conducted by an independent researcher who is blind to the fluoridation status of the subjects. For this study, dental examiners will be trained to take digital oral photographs of the incisor teeth according to a standardised photographic procedure (a modified version of that developed by Cochran *et al*. (2004)). These digital oral photographs of the incisor teeth will be assessed by an experienced researcher at the OHSRC (DB), using the Thylstrup Fejerskov (TF) index ( Thylstrup & Fejerskov, 1978) as modified by Fejerskov *et al* *.* ( Fejerskov *et al*., 1988).

The teeth will first be photographed “wet” to record the appearance of the teeth under normal conditions. This is important from a public health perspective, as the use of photographs can enhance minor defects, particularly if the teeth are dry, and could lead to an overestimate of the prevalence of defects. The teeth will also be photographed “dry” to allow detection of fluorosis at the very lowest levels of severity.

The teeth will be photographed using a Canon EOS 1100D digital camera with a Sigma 105mm macro lens and Sigma EM140-DG macro ring flash for even lighting. The photographs will be taken from approximately 30 degrees above the perpendicular to reduce lip shadow. In practice, this is a subjective assessment and where doubt occurs, the examiner should bear in mind that it is better to over-estimate the camera angle and be slightly steeper. Photographs will be expected to fall between 30 ^o^ and 45 ^o^ above the incisal edge of the tooth. The reproduction will be set at 1:1, or life-size. Further information regarding the method used to photograph the incisor teeth can be found in the fieldwork manuals 2013–2014 and 2016–2017 ( Supplementary File 2).

To avoid any possibility of difficulty identifying the photograph associated with a particular child due to incomplete record keeping or loss of paper-based records, four images will be taken per child: the first and fourth images will be of the child’s barcode from the consent form and the second and third images will be of the child’s teeth. This will ensure that at all times, the image and subject number can be matched simply by sorting all images by the time and date when they were taken.

**DDE (Developmental Defects of Enamel):**

Not all opacities of enamel are fluorotic. It is important to distinguish between developmental defects of enamel that are not typical of fluorosis, and those that are measured using the fluorosis indices, since non-fluorotic opacities could affect the appearance of teeth, and consequently influence perceptions of aesthetic acceptability and quality of life. The DDE index ( Clarkson & O'Mullane, 1989) records the presence of any enamel opacities or defects in the quantity or quality of enamel without attributing a cause.

The index tooth surfaces to be examined using the DDE in this study are the labial surfaces of the maxillary first pre-molar, canine and incisor teeth and the buccal surfaces of the mandibular first permanent molars.

**Molar-incisor hypomineralisation**

Molar-incisor hypomineralisation (MIH) is a developmental condition resulting in enamel defects in first permanent molars and permanent incisors. Enamel defects can range from mild opacities, white or yellow in colour, to severe enamel involvement, which breaks down rapidly shortly after tooth emergence ( Daly & Waldron, 2009). In addition to being of public health importance due to the symptoms associated with it and the complexity of treating the condition, MIH could potentially be confused with the moderate and severe Dean’s Index scores. Therefore examiners will be trained to recognise the condition and to note the presence or absence of MIH in the clinical record.

**Self-perception of enamel opacities**

Measurement of self-perceived enamel opacities will be recorded according to the procedure outlined in the NHS Dental Epidemiology Programme Oral Health Survey of 12-year-old children, 2008–2009 National Protocol ( www.nwph.net/dentalhealth/). The examining teams will be provided with a laminated page showing three sets of photographs, showing groups of teeth with varying types of appearance.

The questions the examiner will ask each 6 ^th^ class child are presented in Table 3.

**Table 3.** Questions to be asked for the measurement of self-perceived enamel opacities in 12 year old participants.

Question	*Response* (entered into the clinical record)
**“Do you have any white marks on your front teeth that won’t brush off?”**	*Yes* *No* *Don’t know*
For those who say **“Yes”,** the examiner then asks: **“Does the appearance of** **these white marks bother you?”**	*Yes* *No* *Don’t know*
The examiner shows three sets of photographs showing groups of teeth with varying types of appearance. **All children** are then asked **“Thinking about white marks on teeth, do you** **think your front teeth look more like those in this group, or the ones in** **this group, or this group?”**	*Photograph set N* *Photograph set S* *Photograph set A* *Don’t know*

**Index of Orthodontic Treatment Need (IOTN)– aesthetic component**

The IOTN has two components which independently measure the need for orthodontic treatment based on aesthetic grounds and on dental health grounds. In the FACCT study, only the aesthetic component will be used to control for impact on quality of life and on perceptions of aesthetics that are due to malocclusion rather than caries or fluorosis. A set of ten special colour photographs will be used to gauge the Aesthetic Component.

To measure the Aesthetic Component of the IOTN:

- The child is seated upright and the examiner views the teeth from in front of the child. The examiner asks the child to close together on their back teeth, then retract the lips to expose the anterior teeth.
- The dental attractiveness is then rated using the 10-point Aesthetic Component scale:
  - **Grades 1–4** represent no need for orthodontic treatment
  - **Grades 5–7** represent a borderline need
  - **Grades 8–10** represent a definite need for orthodontic treatment on aesthetic grounds.
- The anterior teeth should be rated on their dental attractiveness as seen, rather than specific morphological similarity to the photographs. Stained teeth, enamel fractures and gingival inflammation should be ignored.

**Height and weight**

Children’s height will be measured using Leicester Height Measure and weight will be measured using a calibrated Tanita professional weight scales (Tanita, Amsterdam, The Netherlands). Measurements of height and weight will be taken in a sensitive and confidential manner.

*Ensuring consistency of outcome measurement during the study*

To measure intra-examiner agreement, 5% of the children will be examined twice by the same dental examiner for the relevant clinical indices within a 1–7 day interval. The dental examiner will not know, *a priori,* which child is going to be examined twice. Where the initial and repeat examinations are conducted on the same day, examining teams will be advised to allow maximum time between the child’s first examination and the re-examination to minimise recall bias.

Duplicate examinations for dmf/DMF, height and weight will be conducted for all age groups. Additional duplicate measurements will vary depending on the age of the child. Where relevant, duplicate examinations will be conducted for Dean’s Index, DDE Index, IOTN aesthetic component, self-perception of enamel opacities and molar-incisor hypomineralisation. For 5% of subjects, a repeat oral photograph will be taken by the same examiner.

To measure inter-examiner agreement, 5% of the children will be re-examined by a benchmark examiner for all relevant indices. Duplicate examinations will be facilitated by arranging for the benchmark examiner to visit at least one school together with the dental examiner in each study area to examine the children.

Intra-examiner and inter-examiner agreement will be determined using Kappa statistics and percentage agreement (Po). The arbitrary divisions provided by Landis & Koch (1977) will be used as a guide in interpreting the Kappa statistics.

### Classification of fluoridation status

Monthly sample results concerning the level of fluoride in each fluoridated water supply in Dublin, Cork and Kerry will be compiled from the beginning of 2000 (the earliest date of birth of a 6 ^th^ class child in SY 2013–2014). Records will be updated continuously until fieldwork ends in 2017. Maps of distribution networks and data concerning changes to distribution systems will be gathered from engineers, initially up to mid-2014, and again up to mid-2017. Each child’s residential history will be analysed to establish if it meets the criteria for being classified as fluoridated or non-fluoridated; if not, children will be classified as part-fluoridated, or unknown where data are incomplete or where children have lived abroad. A more detailed description of the rules used in classifying the fluoridation status of each child and the rationale for the same can be found in Supplementary File 3

### Data analysis and statistical plan

The impact of CWF on dental caries experience adjusted for the effect of behavioural and socio-demographic factors, will be assessed by conducting multivariate regression analyses using appropriate statistical models. The negative binomial hurdle model (NB-Hurdle), the Poisson Regression Model (PRM) and the Negative Binomial Regression Model (NBRM) will be considered. Model selection will be guided by tests such as the Likelihood-ratio (LR) test, Vuong test, Akaike’s Information Criterion (AIC) and the Bayesian Information Criterion (BIC).

The main explanatory variable in the regression analyses will be the fluoridation status of the child, categorised as ‘fluoridated’, where the child has always resided in an area with CWF, and ‘non-fluoridated’, where the child has never resided in an area with CWF. The analyses will be adjusted for variables such as age, gender, toothbrushing practices, use of toothpaste, whether the child was breastfed, frequency of snacking (sweet foods or drinks), attendance at the dentist, and socioeconomic status.

The impact of CWF on enamel fluorosis adjusted for the effect of behavioural and socio-demographic factors, will be assessed using logistic regression analyses. The analyses will be adjusted for variables such as age, gender, toothbrushing practices, use of toothpaste, whether the child was breastfed and where relevant, type of water used to make up infant formula.

The conventional p-value of < 0.05 will be chosen as the level of significance for all statistical calculations.

Interactions between all explanatory variables will be tested using moderated multiple regression. Only interaction terms significant at the 5% level in moderated multiple regression will be included in the final analyses. Stata SE 10 ( StataCorp, 2007), SAS Version 9 or IBM SPSS Version 23 will be used to analyse the data.

To estimate the impact of oral health on oral health-related quality of life (OHRQoL) of children and their parents the subscale and total scale scores from the validated instruments employed in the study will be correlated with dental caries and enamel fluorosis levels.

The study results will be reported in accordance with the STROBE guidelines for reporting of observational studies ( von Elm *et al*., 2014)

### Dissemination and communication of study results

FACCT results and outcomes will be disseminated to collaborators, policymakers, dental and health professionals and the public, including the school bodies who will be associated with the conduct of FACCT research, through oral presentations, written reports and information leaflets. Presentations including conference proceedings such as slides of study results and scientific posters and leaflets of study results will be posted on the FACCT website (facct.ucc.ie). Key study results will be written up and submitted for publication in peer reviewed journals.

## Data availability

*All data underlying the results are available as part of the article and no additional source data are required.*

## Supplementary material

Supplementary File 1: Zip file of the documents contained in the FACCT consent packs in 2013–2014 and 2016–2017:

- FACCT information leaflet 2013–2014
- FACCT information leaflet 2016–2017
- FACCT consent/assent form 2013–2014
- FACCT consent/assent form 2016–2017
- FACCT residential history form 2013–2014
- FACCT residential history form 2016–2017
- FACCT parent/caregiver questionnaire Junior Infants (age 4–6) 2013–2014
- FACCT parent/caregiver questionnaire 6 ^th^ class (age 11–13) 2013–2014
- FACCT parent/caregiver questionnaire 2 ^nd^ class (age 7–9) 2016–2017

Supplementary File 2: Fieldwork manuals used to assist the examining teams to organise and conduct the oral epidemiological fieldwork:

- Fieldwork Manual 2013–2014
- Fieldwork Manual 2016–2017

Supplementary File 3: The rules that guided the classification of the fluoridation status of each child and the rationale for the classification system used:

- FACCT Fluoridation Status Rules
