# Supplementary material for: Fluoride And Caring for Children’s Teeth (FACCT): Clinical Fieldwork Protocol
Source: HRB Open Res. 2018 Feb 28;1:4. [Version 1] doi: 10.12688/hrbopenres.12799.1 (PMC6973526; doi:10.12688/hrbopenres.12799.1)
Supplement: Supplementary file 3 [file hrbopenres-1-13859-s0002.tgz › 018ae596-e2e1-4544-b320-29c770ab658c.pdf]

## Fluoridation Status Rules

From FACCT Protocol: *CLASSIFICATION OF FLUORIDATION STATUS*

*Monthly sample results concerning the level of fluoride in each fluoridated water supply in Dublin, Cork and Kerry will be compiled from the beginning of 2000 (the likely earliest date of birth of a 6th class child in SY 2013-2014). Records will be updated continuously until fieldwork ends in 2017. Maps of distribution networks and data concerning changes to distribution systems will be gathered from engineers, initially up to mid-2014, and again up to mid-2017. Each child's residential history will be analysed to establish if it meets the criteria for being classified as fluoridated or non-fluoridated; if not, children will be classified as part-fluoridated, or unknown where data are incomplete or where children have lived abroad.*

The following table (Table 1. FACCT Fluoridation Status Rules) will be used to designate individual fluoridation status for each child. The rules are based on considerations relating to the role of fluoride in preventing caries and causing fluorosis. The importance of the timing of exposure to fluoride for the development of both conditions is taken into account and the relevant factors which influenced the final rules are set out in Table 2 (caries) and Table 3 (fluorosis) for information. Only the rules in Table 1 will be used to classify individual fluoridation status.

*Table 1. FACCT Fluoridation Status Rules*

| Fluoridation Status | Description | Age in years | Rules |
|---|---|---|---|
| **FULL** | Lifetime exposure to domestic water fluoridation | 5 | • Must be continuously fluoridated at > 0.4 ppm from at least 6 months old to date of examination<br>  ○ The 1st ≤ 6 months continuous without water fluoridation at > 0.4 ppm is acceptable |
| | | 12 | • Must be continuously fluoridated at > 0.4 ppm from at least 6 months old to at least 6 months before the date of examination<br>  ○ The 1st ≤ 6 months continuous without water fluoridation at > 0.4 ppm is acceptable<br>  ○ The last ≤ 6 months continuous before date of examination without water fluoridation at > 0.4 ppm is also acceptable |
| **NON** | No exposure to domestic water fluoridation | 5 | • Must be continuously non-fluoridated at < 0.4 ppm from at least 6 months old to date of examination<br>  ○ The 1st ≤ 6 months continuous with water fluoridation at > 0.4 ppm is acceptable |
| | | 12 | • Must be continuously non-fluoridated at < 0.4 ppm from at least 6 months old to at least 6 months before the date of examination<br>  ○ The 1st ≤ 6 months continuous with water fluoridation at > 0.4 ppm is acceptable<br>  ○ The last ≤ 6 months continuous before date of examination with water fluoridation at > 0.4 ppm is also acceptable |
| **PART** | Some exposure to domestic water fluoridation, but does not meet criteria for FULL or NON | All | • Does not meet the criteria for FULL because > 6 months continuous without water fluoridation at > 0.4ppm at any age<br>• Does not meet the criteria for NON because > 6 months continuous with water fluoridation at > 0.4ppm at any age<br>• Participants with multiple shorter periods without fluoridation at > 0.4 ppm will be classified on an individual basis considering the timing and duration of the fluctuations of their exposure to domestic water fluoridation |
| **UNKNOWN** | Unknown exposure to domestic water fluoridation | All | • Lived abroad (do not include *holidays* abroad unless > 6 months continuous duration)<br>• Parents refused to complete residential history |
| **• Cases not covered by these rules will be reviewed and classified on an individual basis.** | | | |

**Note to data entry personnel:**

- You **<u>must</u>** refer to the spreadsheets to find out if a supply in Cork or Kerry is <u>continuously</u> fluoridated. Many are not!
- For Dublin, if the supply is fluoridated, you can safely assume it is continuous.

*Table 2. Available data on the relationship between dental caries and water fluoridation suggested that the following definitions are appropriate for categorisation in relation to dental caries in the FACCT sample of 5-year-olds and 12-year-olds, where dental caries in both the primary and permanent dentition will be scored.*

| Fluoridation Status | Age in years | Dental Caries-Related Considerations |
|---|---|---|
| **FULL** | 5 | • Must be continuously fluoridated at $>$ 0.4 ppm from at least 6 months old to date of examination<br>    ○ The 1st $\leq$ 6 months continuous without water fluoridation $>$ 0.4 ppm is acceptable |
| | 12 | • Must be continuously fluoridated at $>$ 0.4 ppm from at least 6 months old to at least 6 months before the date of examination<br>    ○ The 1st $\leq$ 6 months continuous without water fluoridation$>$ at 0.4 ppm is acceptable<br>    ○ The last $\leq$ 6 months continuous before date of examination without water fluoridation$>$ at 0.4 ppm is also acceptable |
| **NON** | 5 | • Must be continuously non-fluoridated at $<$ 0.4 ppm from at least 6 months old to date of examination<br>    ○ The 1st $\leq$ 6 months continuous with water fluoridation at $>$ 0.4 ppm is acceptable |
| | 12 | • Must be continuously non-fluoridated at $<$ 0.4 ppm from at least 6 months old to at least 6 months before date of examination<br>    ○ The 1st $\leq$ 6 months continuous with water fluoridation at $>$ 0.4 ppm is acceptable<br>    ○ The last $\leq$ 6 months continuous before date of examination with water fluoridation at $>$ 0.4 ppm is also acceptable |
| **PART** | All | • Does not meet the criteria for FULL because $>$ 6 months continuous without water fluoridation at $>$ 0.4ppm at any age<br>• Does not meet the criteria for NON because $>$ 6 months continuous with water fluoridation at $>$ 0.4ppm at any age |
| **UNKNOWN** | All | • Lived abroad (do not include *holidays* abroad unless $>$ 6 months continuous duration)<br>• Parents refused to complete residential history |

*Table 3. Data available on the relationship between enamel fluorosis and water fluoridation suggests the following definitions are appropriate in relation to enamel fluorosis in the permanent dentition of the 12-year-old children in the FACCT study*

| Fluoridation Status | Age in years | Enamel Fluorosis-Related Considerations |
|---|---|---|
| **FULL** | 5 | • Must be continuously fluoridated at $> 0.4$ ppm from at least 6 months old to date of examination<br>○ The 1st $\leq$ 6 months continuous without water fluoridation at $> 0.4$ ppm is acceptable |
| | 12 | • Must be continuously fluoridated at $> 0.4$ ppm from at least 6 months old to at least age 7<br>○ The 1st $\leq$ 6 months continuous without water fluoridation at $> 0.4$ ppm is also acceptable |
| **NON** | 5 | • Must be continuously non-fluoridated at $< 0.4$ ppm from at least 6 months old to date of examination<br>○ The 1st $\leq$ 6 months continuous with water fluoridation at $> 0.4$ ppm is acceptable |
| | 12 | • Must be continuously non-fluoridated at $< 0.4$ ppm from at least 6 months old to at least age 7<br>○ The 1st $\leq$ 6 months continuous with water fluoridation at $> 0.4$ ppm is also acceptable |
| **PART** | All | • Does not meet the criteria for FULL because $>$ 6 months continuous without water fluoridation at $>$ 0.4ppm at any age<br>• Does not meet the criteria for NON because $>$ 6 months continuous with water fluoridation at $>$ 0.4ppm at any age |
| **UNKNOWN** | All | • Lived abroad (do not include *holidays* abroad unless $>$ 6 months continuous duration)<br>• Parents refused to complete residential history |
