# Supplementary material for: Fluoride And Caring for Children’s Teeth (FACCT): Clinical Fieldwork Protocol
Source: HRB Open Res. 2018 Feb 28;1:4. [Version 1] doi: 10.12688/hrbopenres.12799.1 (PMC6973526; doi:10.12688/hrbopenres.12799.1)
Supplement: Peer review history

# Peer review: Reviewer response for version 1

**DOI:** 10.21956/hrbopenres.13859.r26075

**Published:** 2018-04-03

**Authors:** Loc G. Do

**Affiliations:** 1 Australian Research Centre for Population Oral Health, University of Adelaide, Adelaide, SA, Australia

The FACCT clinical fieldwork protocol by James et al. described a major population-based oral epidemiological study in Ireland. The study aimed to evaluate the impact and the outcome of the change in community water fluoridation (CWF) policy (2007) on dental caries and enamel fluorosis in Irish schoolchildren, while also considering the change in policy on the use of fluoride toothpastes (2002). The evaluation of several major dental public health policies can be of importance for not only Ireland but also the international dental research community.

 The study is a result of an extensive collaboration between the national and international experts in the field. Data collection instruments and methods are well tested in similar scale research. There is little doubt about feasibility of the study.

 The below comments discuss possible improvements of the study.

- **The study’s focus**. It is believed that the policy changes in 2002 and 2007 were implemented with a desire to lower the prevalence of dental fluorosis in the population without impacting the effectiveness of the preventive measures, namely the use of fluoridated toothpaste and CWF. This focus could be further emphasized, particularly as justification of the study design. The study appears to focus on evaluating the effectiveness of CWF on dental caries using a more recent data. While it is important to do so, it would add little to quantifying ‘the impact of the change’.
  The main characteristic of the study design allows it to be a controlled before-and-after study. Such feature would need to be emphasized. Emphasis in that design would better guide analytical plan and dissemination of the study findings.
- **Analysis to achieve the overall objective**. The specific objectives and analytical plan would need to provide more details on how the evaluation of ‘the impact of the change’ would be undertaken.
  It is believed that identifying and quantifying reduction in the effectiveness of CWF, if any, is at least as important as confirming the effectiveness of CWF at the new level. Such quantification is the most concerning issue for the policy makers and the profession.
- **Population parameter estimates**. It is useful to report how population estimates will be obtained. As the sample was selected through a multistage, stratified sampling frame, weighting procedure is required. Such weighting procedure can also adjust for variations in participation across socioeconomic groups and areas.
  Standardisation of the outcome estimates to population benchmarks at each time point would allow for better comparison.
- **Multilevel analysis**. An alternative analysis possible in this design is multilevel analysis. This approach is desirable given clustering of the outcomes. CWF would then be analysed as a population-level determinant.
- **Difficulty to distinguish between impact of the two policy changes**. Both policy changes have been mentioned in the aim of the study. However, there is a lack of information on how to evaluate effect of the change in policy on the use of fluoride toothpaste. The objective # 4 would provide information on the use of fluoride toothpaste at the current time. There is no mention of how the policy change in 2002 might impact the use of toothpaste.
  It is acknowledged that the study as designed is not able to distinguish possible impact of the two policy changes on dental caries given caries experience is the main outcome. It may be possible to evaluate potential impact of the policy change in 2002 on the prevalence of dental fluorosis. However, such evaluation would require to use dental fluorosis on the incisors as the main outcome. The Dean index as described measures fluorosis experience on any permanent teeth. Hence, the window of susceptibility for fluorosis measured by the Dean is considerably large and wider than the whole gap between the two changes (2002-2007). Using the Dean index may not allow for allocating any change in the prevalence of fluorosis to either of the policy changes. Using fluorosis experience on the incisors only (which have window of susceptibility during the first 3 years of life) may allow for such assessment. It would be done by comparing the prevalence of fluorosis collected for the 12-year old SY 2013-14 (potentially impacted by the 2002 change only) with that for the 8-year old SY 2016-17 (potentially impacted by both changes).
- **Sampling issues**. It is useful to report the targeted numbers of schools for each area. The reported design effect of 1.4 seems small. Dental caries in children tends to cluster within schools and residential areas. Has the design effect been estimated using existing empirical data? Actual statistical power to achieve the aims may need to be estimated while reporting the study findings.
- **Longitudinal analysis**. While this is not one the main aspects of the study, there is an opportunity to conduct longitudinal analysis of individual changes of dental caries from age 5 to 8 years.

I confirm that I have read this submission and believe that I have an appropriate level of expertise to confirm that it is of an acceptable scientific standard.

# Peer review: Reviewer response for version 1

**DOI:** 10.21956/hrbopenres.13859.r26114

**Published:** 2018-04-03

**Authors:** Mark D. Robertson

**Affiliations:** 1 School of Dentistry, University of Dundee, Dundee, UK

Thank you for inviting me to referee the protocol for Fluoride and Caring for Children's Teeth (FACCT). This is an interesting piece of work and I look forward to following FACCT's progress over the next few years. Having read the protocol in detail, I'm happy to offer my approval. 

 FACCT intends to conduct a prospective cross-sectional study (nested longitudinal study) that will both quantify and qualify dental caries experience and dental fluorosis prevalence in school children in the Republic of Ireland who have had variable exposure to community water fluoridation programs and fluoride toothpaste use throughout their lives. The differences in lifetime exposure to fluoride in the study sample is accounted for by the reduction in optimal water fluoridation levels, implemented in 2007, and recommendation from the Forum on Fluoridation in 2002 to avoid use of fluoride containing toothpastes for children under the age of two. Those children aged five years-old at consent (born after introduction of the reduction in water fluoridation levels and recommendations regarding fluoride toothpaste use) will be followed up again at eight years-old to make up the nested longitudinal study. 

 Primarily, FACCT aims to compare the primary oral health outcomes dental caries and enamel fluorosis between the groups. It also intends to estimate the oral health related quality of life for both children and their parents/ carers and to compare the BMI of the participants with that obtained for a similar group in 2002 (Whelton et al., 2006). 

 Methodologically, the FACCT study's protocol is detailed, explicit and clear. Explanation of the participants' representativeness and reasons for which children will be excluded from taking part are also clear, however I wonder if introducing the spread of socio-economic groups included in the study earlier on in the protocol might work better. Perhaps under the 'Participants' subheading within the Methodology section on page 5 of 13. I appreciate this is likely to be fully accounted for by the random sampling of schools throughout the different counties, but I did find myself looking for a statement addressing this before reading the parenthesised statement on page 6 of 13. 

 Additionally, I feel I would find it helpful to have a short explanation of why the height and weight of the participants will be collected to allow for comparison with children from the 2002 North South Survey of Children's Oral Health. Again, I assume this is to consider children's growth and development and how it may be affected by oro-facial pain and/ or infection but I think for a non-dental audience, this would benefit from a little more detail in relation to its relevance. 

 Otherwise, I feel this protocol reads very well and is clear, detailed and scientifically sound. Further, I understand that the full protocol will be a large document and I think the authors have done a brilliant job of condensing the important information and detail into this paper.

 Thank you once again.

 Best wishes,

 Mark Robertson

I confirm that I have read this submission and believe that I have an appropriate level of expertise to confirm that it is of an acceptable scientific standard.
